# Prediction of clinical outcomes after kidney transplantation from deceased donors with acute kidney injury: a comparison of the KDIGO and AKIN criteria

**DOI:** 10.1186/s12882-017-0461-5

**Published:** 2017-01-27

**Authors:** Jeong Ho Kim, Young Soo Kim, Min Seok Choi, Young Ok Kim, Sun Ae Yoon, Ji-Il Kim, In Sung Moon, Bum Soon Choi, Cheol Whee Park, Chul Woo Yang, Yong-Soo Kim, Byung Ha Chung

**Affiliations:** 1 Transplant research center, Seoul, Korea; 2 Division of Nephrology, Department of Internal Medicine, Seoul St. Mary’s Hospital, 505 Banpo-Dong, Seocho-Ku, 137-040 Seoul, Korea; 3 Department of Surgery, Seoul St. Mary’s Hospital, Seoul, Korea; 4 Division of Nephrology, Department of Internal Medicine, Uijeongbu St. Mary’s Hospital, College of Medicine, The Catholic University of Korea, Seoul, Korea

**Keywords:** Acute kidney injury (AKI), Deceased donor (DD), Kidney transplantation (KT), KDIGO, AKIN

## Abstract

**Background:**

Acute kidney injury (AKI) is frequently detected in deceased donors (DDs), and it could be associated with adverse clinical outcomes in corresponding kidney transplant recipients (KTRs). In this regard, we sought to identify which criteria is better between the KDIGO and AKIN criteria for the diagnosis of AKI in DDs in the prediction of clinical outcomes after kidney transplantation (KT).

**Methods:**

Two hundred eighty-five cases of deceased donor kidney transplantation (DDKT) were included. We divided them into three groups; the non-AKI by both KDIGO and AKIN criteria group (*n* = 120), the AKI by KDIGO only group (*n* = 61), and the AKI by both criteria group (*n* = 104) according to the diagnosis of AKI using the KDIGO and AKIN criteria in the corresponding 205 DDs. We compared the development of delayed graft function (DGF), the change in allograft function, the allograft survival among the three groups.

**Results:**

The incidence of DGF was significantly higher in the AKI by KDIGO only and the AKI by both criteria groups than in the non-AKI by both criteria group (*P* < 0.05 each). But no difference was detected between the AKI by KDIGO only group and the AKI by both criteria group (*P* > 0.05). Therefore, the KDIGO criteria had a better predictive value for DGF occurrence than the AKIN criteria (Area under the curve = 0.72 versus 0.63, *P* < 0.05) in Receiver Operation Characteristic analysis. On comparison of allograft function, the AKI by KDIGO only and the AKI by both criteria groups showed a significantly deteriorating pattern by 6 months after KT in comparison with the non-AKI by both criteria group (*P* < 0.05). However, the differences disappeared at 1 year from KT and long-term allograft survival did not differ among the three groups. AKI stage either by KDIGO or AKIN in DDs did not affect long-term allograft survival in corresponding KTRs as well.

**Conclusions:**

The KDIGO criteria may be more useful for predicting DGF than the AKIN criteria. However, AKI or AKI stage by either criteria in DDs failed to affect long-term allograft outcomes in KTRs.

## Background

The imbalance between donors and recipients of kidney transplantation (KT) promoted the introduction of various strategies to increase the potential donor pool for transplantation [1, 2]. In this regard, the use of kidneys from deceased donors (DDs) with acute kidney injury (AKI) has been proposed as an important strategy to solve donor shortage [3–10]. However, specific guidelines or standardized classification methods to determine the severity of AKI in deceased donors have not been established, even though the use of kidneys from DDs with AKI may induce adverse post-transplant outcomes, for example higher incidence of delayed graft function (DGF) recovery [11–13].

Meanwhile, standardized classification systems that represent the status of AKI, have been developed and widely applied in the clinical practice [14]. Initially, the RIFLE (Risk, Injury, Failure, Loss, End-Stage Kidney Disease) criteria were developed by the Acute Dialysis Quality Initiative group and a modification of the RIFLE criteria, known as the AKIN (AKI Network) classification system, was proposed later [15, 16]. More recently, the KDIGO (Kidney Disease: Improving Global Outcomes) proposed a new definition and classification of AKI based on both the RIFLE and AKIN criteria, and it showed a better prediction of the prognosis of AKI in hospitalized patients [17, 18].

Previously, we found that application of the AKIN criteria for diagnosis of AKI in DDs was useful to predict the development of delayed graft function (DGF) recovery in the corresponding kidney transplant recipients (KTRs) [5]. However, the KDIGO criteria, which showed better performance compared to the AKIN criteria in the general population, has not been adopted in this field till now. In this regard, we used the KDIGO criteria for the diagnosis of AKI in DDs, and compared its performance in the prediction of DGF or post-transplant outcomes in corresponding KTRs with the AKIN criteria.

## Methods

### The aim and study populations of the study

**Fig. 1:**
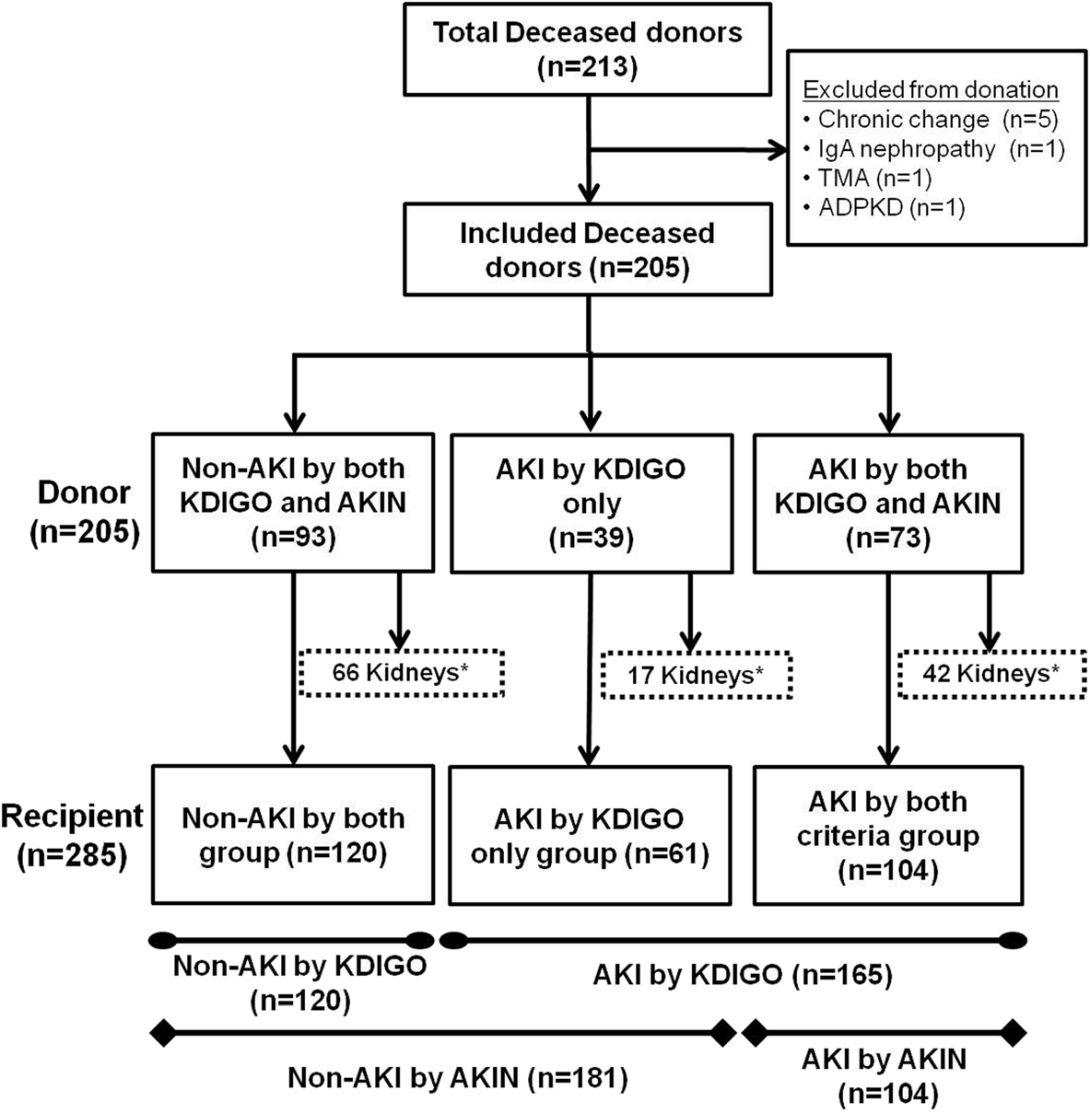
Patient algorithm and distribution in this study. Out of the 213 DDs, 8 cases were excluded because they were not suitable for kidney donation. The remaining 205 cases were divided into three groups; the non-AKI by both KDIGO and AKIN criteria group, the AKI by KDIGO only group, and the AKI by both KDIGO and AKIN criteria group. Out of the 410 kidneys harvested from these DDs, 125 kidneys were transferred to another institution, and 285 kidneys were transplanted in our institutions. Each KTR also belonged to one of the three groups (the non-AKI by both criteria group, the AKI by KDIGO only group, and the AKI by both criteria group) according to the group of corresponding DDs. TMA, thrombotic microangiopathy; ADPKD, autosomal dominant polycystic kidney disease; AKI, acute kidney injury; KDIGO, kidney disease: improving global outcome; AKIN, acute kidney injury network; KTRs, kidney transplant recipients, DDs, deceased donors. *Transferred to another institution by the rule of organ distribution in Korea

The aim of this study is to identify which criteria are better between the KDIGO and AKIN criteria for the diagnosis of AKI in DDs in the prediction of clinical outcomes after KT. We included deceased donor kidney transplantation (DDKT) performed at Seoul St. Mary’s hospital and Uijeongbu St. Mary’s hospital between September 1996 and March 2014. During this period, 426 kidneys were harvested from 213 DDs in Seoul St. Mary’s hospital. Among them, 16 kidneys from 8 potential donors were discarded because they were not found to be suitable for transplantation on donor kidney biopsy (5 cases of advanced chronic change, 1 case of IgA nephropathy, 1 case of thrombotic microangiopathy) or because of underlying disease (1 case of autosomal dominant polycystic kidney disease). Therefore, 410 kidneys from 205 DDs were used for KT. Among them, 125 kidneys were transferred to another institution for KT according to the organ distribution rule in Korea. Finally, 285 kidneys were transplanted in Seoul St. Mary’s hospital (*n* = 265) or Uijeongbu St. Mary’s hospital (*n* = 20). We included these 205 donors and 285 corresponding KTRs in this analysis (Fig. 1).

### Diagnosis of AKI in DDs

According to the KDIGO or AKIN criteria, we determined the stage or severity of AKI in 205 DDs as described in previous reports [16, 19]. Briefly, according to the KDIGO criteria, stage 1 encompasses a serum creatinine (SCR) level increase of ≥0.3 mg/dL within 48 h or increase in SCR to ≥1.5 times baseline, which is known or presumed to have occurred within 7 days, or a reduction in urine output (<0.5 mL/kg/h for 6 h); stage 2, increase in SCR to 2.0-2.9 times baseline or a reduction in urine output (<0.5 mL/kg/h for 12 h); stage 3, increase in SCR to 3.0 times baseline or to ≥4.0 mg/dL, or receipt of renal replacement therapy (RRT) or a reduction in urine output (<0.3 mL/kg/h for 24 h or anuria for 12 h). According to the AKIN criteria, stage 1 is defined as an absolute SCR increase of ≥ 0.3 mg/dL or increase to ≥ 1.5-2 times or a reduction in urine output (<0.5 mL/kg/h for 6 h); stage 2, increase in SCR to >2-3 times or a reduction in urine output (<0.5 mL/kg/h for 12 h); stage 3, increase in SCR to >3 times or to ≥4.0 mg/dL with an acute increase of at least 0.5 mg/dL or receipt of RRT or a reduction in urine output (<0.3 mL/kg/h for 24 h or anuria for 12 h). Two values of SCR within 48 h were used in all AKIN criteria stages.

### Classification of donors and recipients according to the AKIN or KDIGO criteria

Figure 1 shows the distribution of DDs and corresponding KTRs according to the diagnosis of AKI by the KDIGO or AKIN criteria. Out of the 205 DDs, 93 cases (45.4%) were diagnosed as non-AKI and 73 donors (35.6%) were diagnosed as AKI by both the KDIGO and AKIN criteria. However, 39 cases (19.0%) were diagnosed as AKI by the KDIGO criteria, but they did not meet the definition of AKI according to the AKIN criteria. After excluding the 125 kidneys that were transferred to other institutions, 120 patients received kidneys from donors diagnosed with non-AKI by both criteria (the non-AKI by both criteria group) and 104 patients received kidneys from donors diagnosed with AKI by both criteria (the AKI by both criteria group). The remaining 61 patients received kidneys from donors diagnosed with AKI by KDIGO only (the AKI by KDIGO only group). Among these three groups, we compared the baseline characteristics of donors as well as those of recipients (Tables 1, and 2).

**Table 1:** Baseline Characteristics of Donors

	Non-AKI by both(*n* = 93)	AKI by KDIGO only (*n* = 39)	AKI by both(*n* = 73)	*P* value (AKI by KDIGO only vs. Non-AKI by both)	*P* value (AKI by both vs. Non-AKI by both)	*P* value (AKI by KDIGO only vs. AKI by both)
Male	60 (64.5)	27 (69)	52 (71)	0.6	0.4	0.8
Age (years)	40.2 ± 15.6	44.5 ± 14.1	46.3 ± 12.1	0.1	0.005	0.5
BMI (kg/m^2^)	22.2 ± 3.9	24.6 ± 4.0	22.8 ± 3.0	0.005	0.3	0.02
Hypertension	16 (17.2)	6 (15.4)	14 (19)	0.8	0.7	0.6
DM	4 (4.3)	1 (2.6)	8 (11.0)	0.9	0.1	0.1
Urine output (ml/day)ª	4771.6 ± 3625.9	4840.8 ± 3647.3	4082.1 ± 3335.1	0.9	0.2	0.3
Cause of death						
CVA	52 (56)	23 (59)	42 (58)	0.7	0.8	0.9
Trauma	26 (28.0)	12 (31)	23 (32)	0.7	0.6	0.9
Others	15 (16)	4 (10)	8 (11.0)	0.4	0.3	0.9
CVP (cmH_2_O)ª	6.9 ± 3.7	7.3 ± 3.7	7.3 ± 3.6	0.6	0.5	0.9
MAP (mmHg)ª	88.1 ± 14.8	92.9 ± 17.8	88.5 ± 16.3	0.1	0.9	0.2

*Note*: values for categorical variables are given as number (percentage); for continuous variables, as mean ± standard deviation

*Abbreviations*: *AKI* acute kidney injury, *KDIGO* kidney disease: improving global outcomes, *AKIN* acute kidney injury network, *BMI* body mass index, *DM* diabetes mellitus, *CVA* cerebrovascular accident, *CVP* central venous pressure, *MAP* mean arterial pressure

ªDuring the last 24 h before kidney transplantation

**Table 2:** Baseline Characteristics of Recipients

	Non-AKI by both(*n* = 120)	AKI by KDIGO only (*n* = 61)	AKI by both (*n* = 104)	*P* value (AKI by KDIGO only vs. Non-AKI by both)	*P* value (AKI by both vs. Non-AKI by both)	*P* value (AKI by KDIGO only vs. AKI by both)
Male	66 (55.0)	37 (61)	53 (51.0)	0.5	0.5	0.2
Age (years)	46.5 ± 8.0	48.0 ± 9.8	49.1 ± 11.3	0.3	0.05	0.5
Retransplant	13 (10.8)	8 (13)	8 (7.7)	0.7	0.4	0.3
BMI (kg/m^2^)	22.7 ± 3.3	22.8 ± 3.0	22.7 ± 3.6	0.8	0.9	0.9
Primary renal disease						
DM	12 (10.0)	11 (18)	16 (15.4)	0.1	0.2	0.7
Hypertension	24 (20.0)	13 (21)	34 (32.7)	0.8	0.03	0.1
GN	55 (45.8)	23 (38)	33 (31.7)	0.3	0.03	0.4
Others	29 (24.2)	14 (23)	21 (20.2)	0.9	0.5	0.7
Duration of dialysis (years)	7.1 ± 4.9	6.0 ± 4.1	6.6 ± 4.2	0.1	0.4	0.4
PRA (%)	13.1 ± 26.3	15.6 ± 29.6	12.3 ± 26.6	0.6	0.8	0.5
HLA MN	3.9 ± 1.1	3.8 ± 0.9	4.0 ± 1.3	0.6	0.4	0.2
Cold ischemic time (minutes)	193.2 ± 86.5	201.7 ± 108.7	230.8 ± 121.9	0.6	0.01	0.1
Induction therapy				0.2	0.01	0.4
ATG	10 (8.3)	9 (15)	21 (20.2)			
Basiliximab	110 (91.7)	52 (85)	83 (79.8)			
Immunosuppressant						
Cyclosporine	18 (15.0)	11 (18)	9 (8.7)	0.6	0.1	0.08
Tacrolimus	101 (84.2)	50 (82)	95 (91.3)	0.7	0.1	0.08
Sirolimus	1 (0.8)	0 (0)	0 (0)	0.9	0.9	

*Note*: values for categorical variables are given as number (percentage); for continuous variables, as mean ± standard deviation

*Abbreviations*: *AKI* acute kidney injury, *KDIGO* kidney disease: improving global outcomes, *AKIN* acute kidney injury network, *BMI* body mass index, *DM* diabetes mellitus, *GN* glomerulonephritis, *PRA* panel reactive antibody, *HLA* human leukocyte antigen, *MN* mismatch number, *ATG* anti-thymocyte globulin

In addition, we performed two group analysis between the AKI group and the non-AKI group according to the diagnosis of AKI by the KDIGO or AKIN criteria, respectively. Therefore, in the two group analysis based on the KDIGO criteria, the AKI group included 165 patients (the AKI by both criteria plus the AKI by KDIGO only groups), and 120 patients belonged to the non-AKI group (the non-AKI by both criteria group). Based on the AKIN criteria, only 104 KTRs (AKI by both criteria) belonged to the AKI group and the remaining 181 KTRs (the non-AKI by both criteria plus the AKI by KDIGO only groups) belonged to the non-AKI group (Fig. 1).

### Clinical parameters and outcomes

We retrospectively reviewed the medical records of all patients and collected baseline data of the donors including age, sex, body mass index (BMI) (kg/m^2^), history of diabetes mellitus and hypertension, cause of death, last-day urine volume, central venous pressure and mean arterial pressure from the day of admission to the day of KT. In addition, we collected the baseline data of the recipients: age, sex, BMI, primary renal disease, duration of dialysis, number of previous KT, percentage of panel-reactive antibodies (PRAs), number of human leukocyte antigen (HLA) mismatches, type of induction therapy, maintenance immune suppressants, and cold ischemic time.

The primary outcome of this study was the incidence of DGF in KTRs according to the diagnosis of AKI by the KDIGO or AKIN criteria in corresponding DDs. DGF was defined as dialysis requirement within the first week after KT [20]. Secondary outcome included the allograft function during post-transplant 1 year (3 days, 1 week, 2 weeks, 1 month, 3 months, 6 months, and 1 year after KT) determined as the estimated glomerular filtration rate (eGFR) using the modification of diet in renal disease (MDRD) equation [21], and long-term allograft survival rates.

We compared the clinical outcomes in two group analysis between the AKI group and the non-AKI group by the KDIGO or AKIN criteria, respectively, and we also compared these outcomes among the three groups; non-AKI by both criteria; AKI by KDIGO only, and AKI by both criteria.

### Statistical methods

Statistical analyses were performed by using PASW Statistics for Windows, Version 18 (SPSS Inc., Chicago, IL, USA). Data are presented as mean ± standard deviation (SD), or counts and percentages, depending on the data type. The comparison between the AKI and non-AKI groups was analyzed using the Student *t* test or One-way ANOVA test for numerical values and the χ2 test for categorical data. All continuous variables were tested for normal distribution using the Shapiro-Wilk test and were expressed as the mean ± SD. Categorical variables are presented as the percentage of the number of cases. Receiver Operation Characteristic (ROC) analysis was used to calculate the predictability of each AKI criteria for the development of DGF in KTRs. We used a non-parametric test, the Mann-Whitney U test, for comparison of allograft function assessed by the MDRD equation. After univariate analysis of the risk factors for DGF, significant variables were analyzed by multivariate logistic regression analysis. Allograft survival rates were calculated using Kaplan-Meier estimates and patient death was censored in this analysis. Differences between survivals were calculated by log-rank analysis. Significant variables for allograft survival were analyzed by the Cox regression hazard model. *P* < 0.05 was considered statistically significant.

## Results

### Comparison between the KDIGO and AKIN Criteria for the Detection of AKI Severity in DDs

**Fig. 2:**
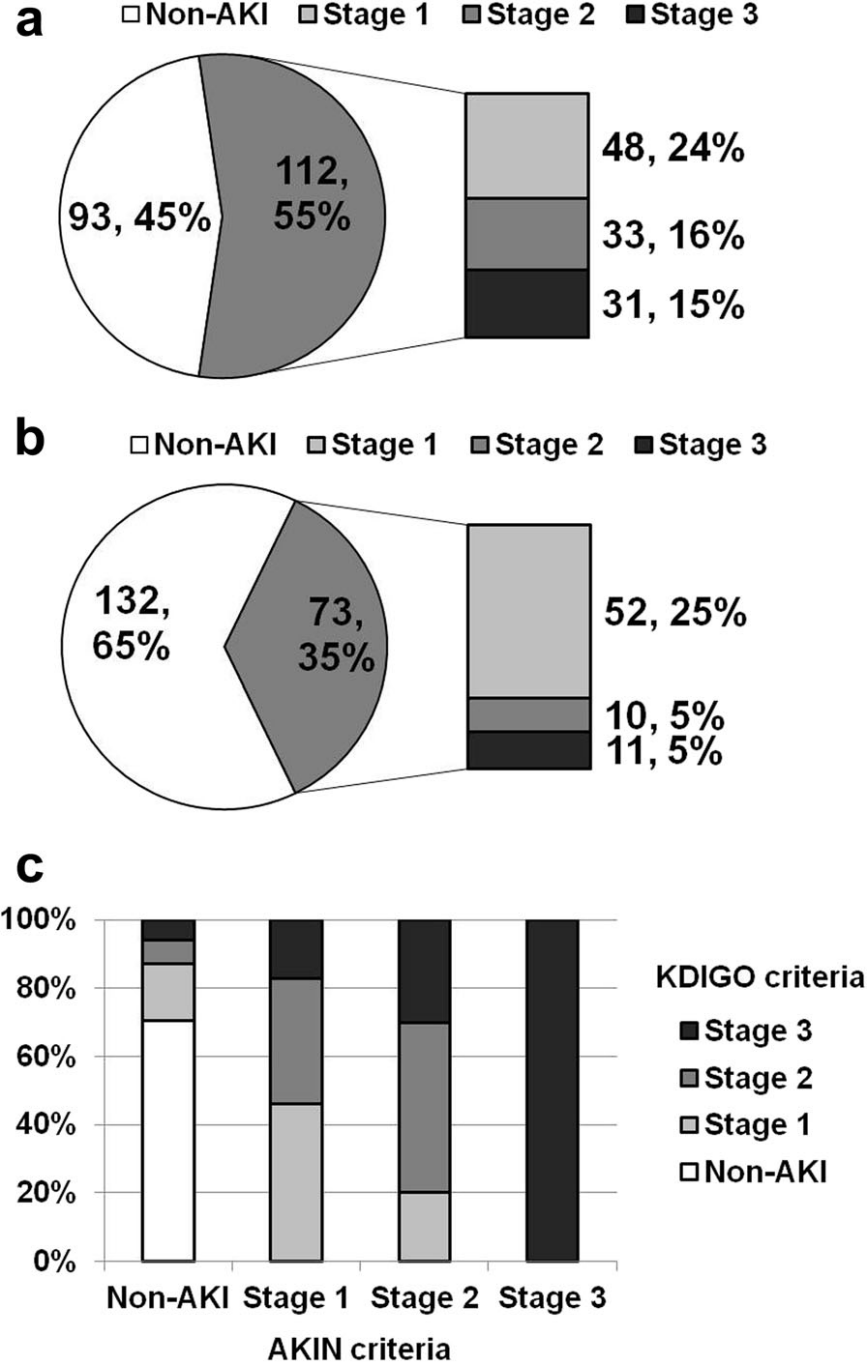
Diagnosis of AKI or AKI stage in DDs according to the (**a**) KDIGO or (**b**) AKIN criteria. Please note that 39 donors belonging to the AKI by KDIGO only group as shown in Fig. 1 belonged to the non-AKI group in patient distribution by the AKIN criteria; hence the proportion of AKI was higher in the donor distribution according to the KDIGO criteria in comparison with the AKIN criteria. **c** The AKI stage in KDIGO showed a significant association with that in the AKIN criteria (Pearson’s correlation coefficient; 0.669, *p* < 0.001). However, there was discordance between these two criteria in 35.1% (72/205) of the total DDs. AKI, acute kidney injury; DDs, deceased donors; KDIGO, kidney disease: improving global outcome; AKIN, acute kidney injury network

Out of the 112 cases (54.6%) of AKI diagnosed by the KDIGO criteria, 42.8% (48/112) cases were stage 1, 29.5% (33/112) cases were stage 2, and 27.7% (31/112) cases were stage 3 (Fig. 2a). According to the AKIN criteria, out of the 73 (35%) cases of AKI, 71.2% (52/73) cases were stage 1, 13.7% (10/73) cases were stage 2, and 15.1% (11/73) cases were stage 3 (Fig. 2b). Figure 2c shows a significant association between KDIGO and AKIN in regard to the distribution of AKI stage (Pearson’s correlation coefficient; 0.669, *p* < 0.001). However, discrepancy between the two criteria was detected in 35.1% (72/205) of cases. Out of the 132 non-AKI donors by AKIN, 16.7% (*n* = 22) donors were stage 1, 6.8% (*n* = 9) donors were stage 2, and 6.1% (*n* = 8) donors were stage 3 AKI according to the KDIGO criteria. Out of the 52 stage 1 donors by AKIN, 36.5% (*n* = 19) donors were stage 2 and 17.3% (*n* = 9) donors were stage 3 according to the KDIGO criteria. Among the 10 stage 2 donors by AKIN, 2 donors were stage 1 and 3 donors were stage 3. All stage 3 donors *(n* = 16) by AKIN were also defined as stage 3 by the KDIGO criteria.

### Comparison between the KDIGO and AKIN criteria for the prediction of the development of DGF

**Fig. 3:**
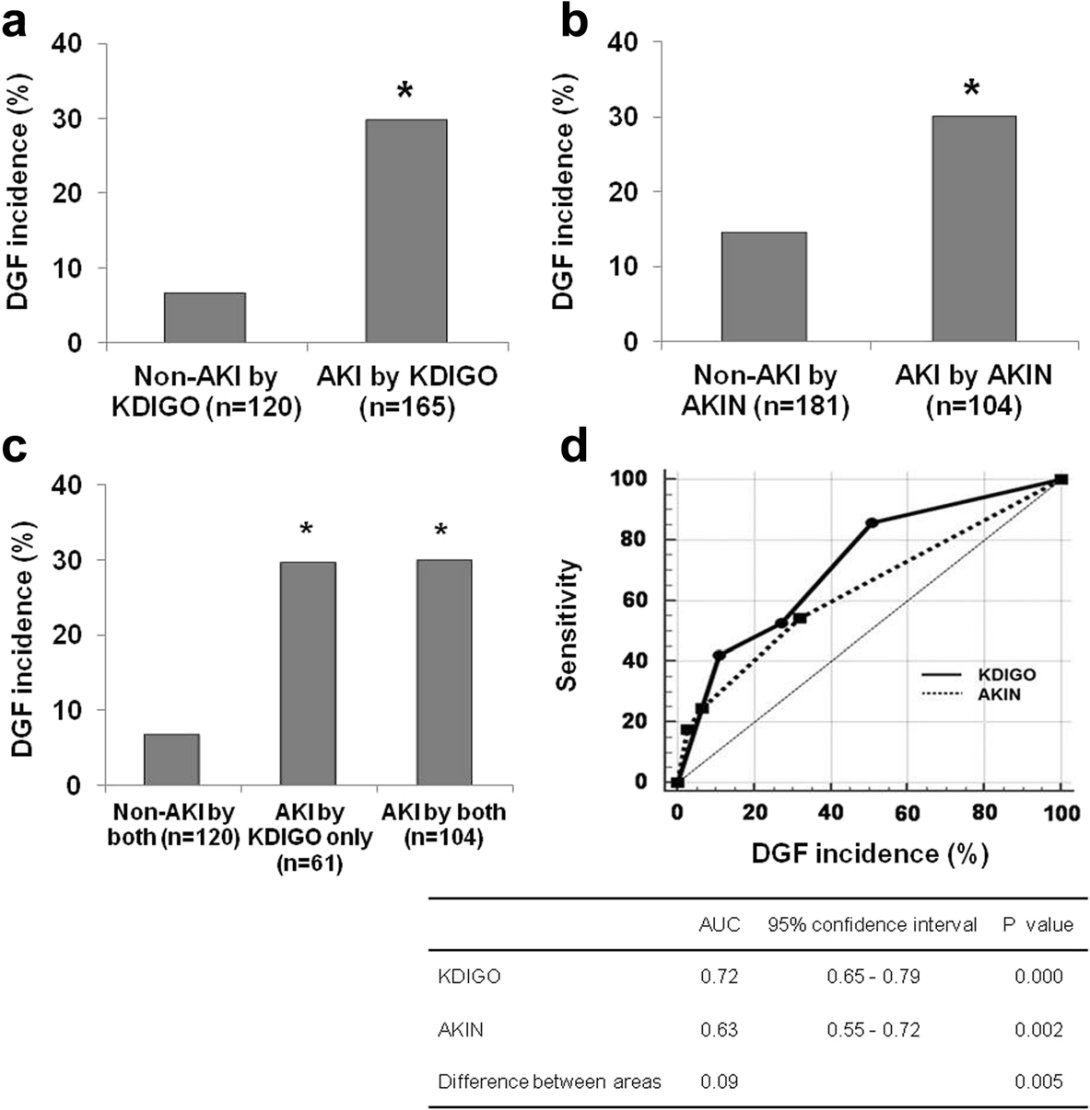
Comparison of the development of DGF between the AKI and non-AKI groups according to the diagnosis of AKI by either the (**a**) KDIGO or (**b**) AKIN criteria in corresponding DDs. Please note that the incidence of DGF was significantly higher in the AKI group irrespective of the criteria applied for the diagnosis of AKI in DDs. **c** Comparison of DGF among the three groups; non-AKI by both criteria, AKI by KDIGO only, and AKI by both criteria groups. Please note that the incidence of DGF in the AKI by KDIGO only group was very similar to that in the AKI by both criteria group, and it was significantly higher than that in the non-AKI by both criteria group. **d** Comparison of the predictive value for DGF between the KDIGO and AKIN criteria. Please note that the AUC was significantly larger in KDIGO (0.72) in comparison with the AKIN criteria (0.63). DGF, delayed graft function; AKI, acute kidney injury; DDs, deceased donors; KDIGO, kidney disease: improving global outcome; AKIN, acute kidney injury, DDKT, deceased donor kidney transplantation; AUC, area under the curve. *P < 0.05 compared with non-AKI group

DGF developed in 57 out of the 285 patients; hence the incidence of DGF was 20%. In the analysis using the KDIGO criteria for the diagnosis of AKI in DDs, DGF developed more frequently in the AKI group than in the non-AKI group (29.7% versus 6.7%; *P* < 0.05; Fig. 3a), and also in another analysis using the AKIN criteria, the incidence of DGF was significantly higher in the AKI group than in the non-AKI group (29.8% versus 14.4%; *P* < 0.05; Fig. 3b). When we compared the development of DGF among the three groups (non-AKI by both criteria, AKI by KDIGO only, and AKI by both criteria groups), the incidence of DGF was significantly lower in the non-AKI by both criteria group (6.7% (8/120)) in comparison with the AKI by KDIGO group (29.5% (18/61)) or the AKI by both criteria group (29.8% (31/104)) (*P* < 0.001 for each). However, it did not show any significant difference between the AKI by KDIGO only and the AKI by both criteria groups (*P* = 1.0) (Fig. 3c).

### Comparison between the KDIGO and AKIN criteria for the prediction of DGF

We investigated whether the diagnosis of AKI by KDIGO or AKIN in DDs can significantly predict the development of DGF in corresponding KTRs. In univariate analysis, male donor, and AKI by the KDIGO criteria and AKI by the AKIN criteria in DDs were significant risk factors for the development of DGF in corresponding KTRs. In multivariate analysis, diagnosis of AKI by the KDIGO or AKIN criteria in DDs was still an independent risk factor for the development of DGF in corresponding KTRs (Table 3). For making a comparison of the predictive value for DGF in KTRs, we performed ROC curve analysis. Finally, the KDIGO criteria showed better prognostic accuracy in the prediction of the development of DGF compared to the AKIN criteria (area under the curve = 0.721 versus 0.636; *P* = 0.01, z statistics; 2.466, Fig. 3d).

**Table 3:** Risk Factor for the Development of Delayed Graft Function

	Univariate		Multivariate	
	Odd ratio (95% CI)	*P* value	Odd ratio (95% CI)	*P* value
Donor				
Age	1.00 (0.98-1.02)	0.7		
Male	3.42 (1.60-7.30)	0.002	2.30 (0.83-6.37)	0.1
BMI	1.08 (0.98-1.18)	0.1		
DM	1.07 (0.34-3.36)	0.9		
Hypertension	1.25 (0.61-2.58)	0.5		
Cause of death (CVA)	1.14 (0.63-2.08)	0.7		
AKI by KDIGO	5.86 (2.37-14.47)	<0.001	6.67 (1.60-27.73)	0.009
AKI by AKIN	5.95 (2.59-13.65)	<0.001	8.93 (2.46-32.44)	0.001
CVP	1.08 (0.99-1.17)	0.08		
MAP	1.01 (0.99-1.03)	0.2		
Last day urine output	1.00 (1.00-1.00)	0.6		
Cold ischemic time	1.00 (1.00-1.00)	0.2		
Recipient				
Age	0.98 (0.96-1.01)	0.3		
Male	1.07 (0.60-1.93)	0.8		
BMI	1.01 (0.92-1.10)	0.9		
DM	1.03 (0.48-2.22)	0.9		
Hypertension	1.34 (0.53-3.38)	0.5		
GN	1.29 (0.72-2.32)	0.4		
Retransplantation	1.61 (0.67-3.85)	0.3		
PRA	1.01 (1.00-1.02)	0.2		
HLA MN	1.19 (0.91-1.55)	0.2		
ATG (Reference = Basiliximab)	0.83 (0.35-1.98)	0.7		
Tacrolimus (Reference = Cyclosporine)	0.23 (0.13-0.48)	<0.001	0.07 (0.01-1.01)	0.06

*Abbreviations*: *CI* confidence interval, *BMI* body mass index, *DM* diabetes mellitus, *CVA* cerebrovascular accident, *AKI* acute kidney injury, *KDIGO* kidney disease: improving global outcome, *AKIN* acute kidney injury network, *CVP* central venous pressure, *MAP* mean arterial pressure, *GN* glomerulonephritis, *PRA* panel reactive antibody, *HLA* human leukocyte antigen, *MN* mismatch number, *ATG* anti-thymocyte globulin

### Comparison of the change in allograft function during post-transplant 1 year

**Fig. 4:**
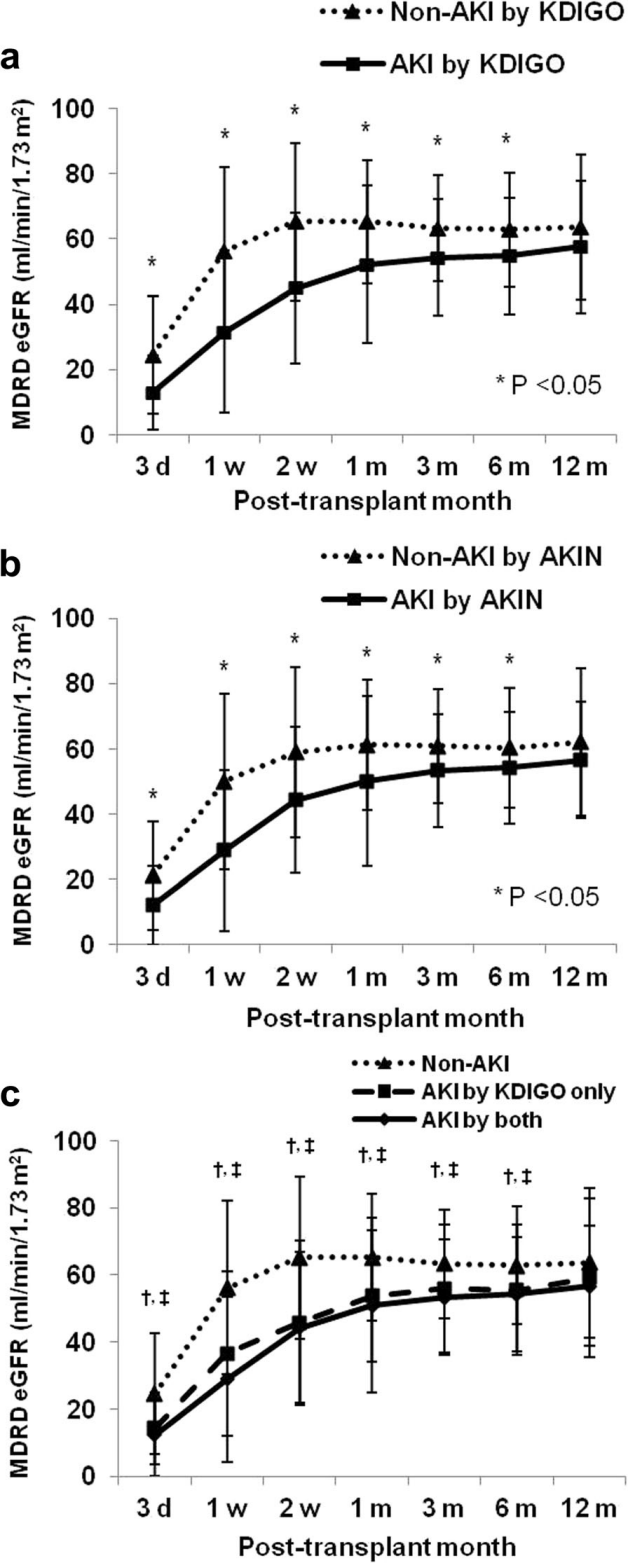
Comparison of the change in allograft function after kidney transplantation between the non-AKI and AKI groups according to the diagnosis of AKI by the (**a**) KDIGO or (**b**) AKIN criteria in corresponding DDs. Please note that allograft function was significantly lower in the AKI group than in the non-AKI group by 6 months after KT, but these differences disappeared at 1 year after KT for any AKI criteria. **c** Comparison of the change in allograft function among the three groups; non-AKI by both criteria, AKI by KDIGO only, and AKI by both criteria groups. Please note that the changing pattern of allograft function in the AKI by KDIGO only group was very similar to that in the AKI by both criteria group. MDRD, the modification of diet in renal disease; eGFR, estimated glomerular filtration rate; AKI, acute kidney injury; DDs, deceased donors; KDIGO, kidney disease: improving global outcome; AKIN, acute kidney injury network; KT, kidney transplantation; d, day; m, month. ^*^P < 0.05 compared with non-AKI group, ^†^P < 0.05 AKI group by KDIGO only compared with non-AKI group, ^‡^P < 0.05 AKI group by KDIGO and AKIN compared with non-AKI group

Figure 4 shows the comparison of the changing pattern of allograft function assessed by the MDRD equation during post-transplant 12 months between the AKI group and the non-AKI group. At each time-point upto 6 months from KT, allograft function was significantly lower in the AKI group in comparison with the non-AKI group in both analyses using the KDIGO or AKIN criteria in DDs. However, these differences in allograft function between the AKI and non-AKI groups showed a diminishing pattern at 12 months from KT in each analysis (Fig. 4a, b). On comparison among the three groups, the non AKI by both criteria group showed a superior allograft function compared to the other 2 AKI groups (AKI by KDIGO only and AKI by both criteria groups) upto 6 months from KT. But, this difference also disappeared at 12 months from KT. Meanwhile, the AKI by KDIGO only and the AKI by both criteria groups showed a totally similar pattern to each other through post-transplant 1 year from KT (Fig. 4c).

### Comparison of allograft survival according to the diagnosis of AKI in corresponding DDs

**Fig. 5:**
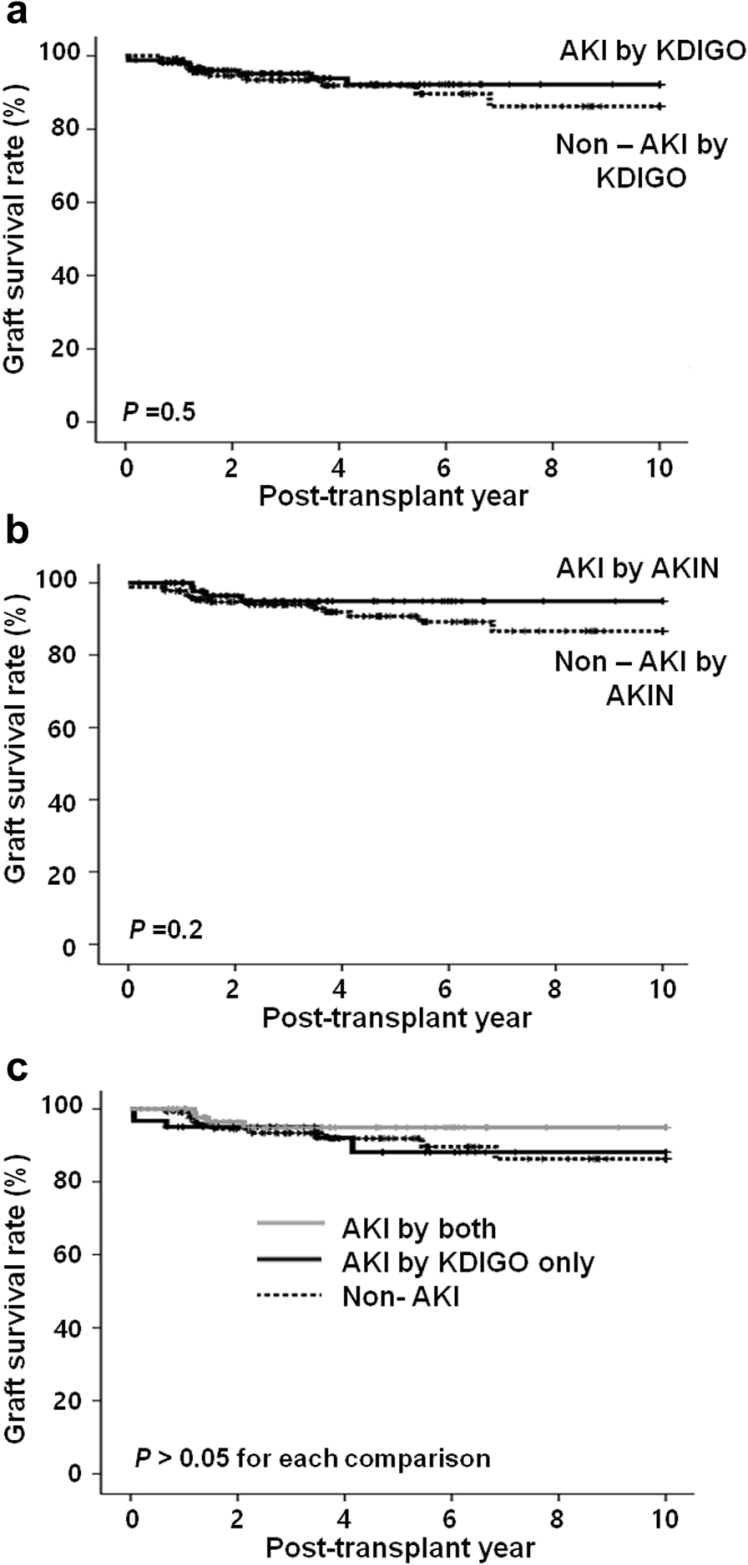
Comparison of the long-term allograft survival rate between the non-AKI and AKI groups according to the diagnosis of AKI by the (**a**) KDIGO or (**b**) AKIN criteria in corresponding DDs. Please note that no difference was detected in allograft survival rate between the AKI group and the non-AKI group in both analyses. **c** On comparison of long-term allograft survival rate among the three groups; non-AKI by both criteria, AKI by KDIGO only, and AKI by both criteria groups, no difference was found. AKI, acute kidney injury; DDs, deceased donors; KDIGO, kidney disease: improving global outcome; AKIN, acute kidney injury network

The long-term allograft survival rate upto 10 years from KT did not differ significantly between the AKI and non-AKI groups in both analyses using the KDIGO (Fig. 5a) or AKIN criteria (Fig. 5b) (*P* = 0.2, *P* = 0.5, respectively). On comparison among the three groups (Non-AKI by both criteria, AKI by only KDIGO, and AKI by both criteria groups), no significant difference was detected (*P* > 0.05 for each comparison, Fig. 5c). In the Cox regression hazard model, allograft survival was independently influenced by BMI of KTRs (OR, 0.57; 95% CI, 0.41 to 0.80; *P* = 0.001), development of DGF (OR, 9.25; 95% CI, 1.93 to 44.33; *P* = 0.005), and development of acute rejection in KTRs (OR, 6.00; 95% CI, 1.06 to 34.01; *P* = 0.04). However, the development of AKI defined as the KDIGO or AKIN criteria in DDs did not show a significant association with allograft survival in corresponding KTRs (Table 4).

**Table 4:** Risk Factor for Death Censored Graft Failure

	Univariate		Multivariate	
	Odd ratio (95% CI)	*P* value	Odd ratio (95% CI)	*P* value
Donor				
Age	1.00 (0.97-1.03)	0.9		
Male	1.15 (0.42-3.11)	0.8		
BMI	0.89 (0.75-1.06)	0.2		
DM	0.77 (0.09-6.06)	0.8		
Hypertension	2.21 (0.80-6.11)	0.1		
Cause of death (CVA)	1.88 (0.66-5.39)	0.2		
AKI by KDIGO	0.98 (0.32-3.01)	0.9	1.37 (0.19-10.09)	0.8
AKI by AKIN	0.44 (0.13-1.45)	0.2	0.18 (0.02-1.40)	0.1
CVP	1.02 (0.89-1.16)	0.1		
MAP	0.97 (0.94-1.01)	0.8		
Last day urine output	1.00 (1.00-1.00)	0.4		
Cold ischemic time	1.00 (0.99-1.00)	0.4		
Recipient				
Age	0.97 (0.92-1.02)	0.2		
Male	1.15 (0.45-2.94)	0.8		
BMI	0.83 (0.70-0.99)	0.03	0.57 (0.41-0.80)	0.002
DM	0.55 (0.12-2.45)	0.4		
Hypertension	1.29 (0.29-5.82)	0.7		
GN	1.45 (0.57-3.68)	0.4		
Retransplantation	1.04 (0.23-4.75)	0.9		
PRA	0.96 (0.91-1.02)	0.2		
HLA MN	1.64 (1.03-2.62)	0.04		
ATG (Reference = Basiliximab)	0.71 (0.16-3.18)	0.6		
Tacrolimus (Reference = Cyclosporine)	0.40 (0.14-1.21)	0.1		
Delayed graft function	1.95 (0.71-5.37)	0.2	9.25 (1.93-44.33)	0.005
Acute rejection	7.55 (2.86-19.91)	<0.001	6.00 (1.06-34.09)	0.04

*Abbreviations*: *CI* confidence interval, *BMI* body mass index, *DM* diabetes mellitus, *CVA* cerebrovascular accident, *KDIGO* kidney disease: improving global outcome, *AKIN* acute kidney injury network, *CVP* central venous pressure, *MAP* mean arterial pressure, *GN* glomerulonephritis, *PRA* panel reactive antibody *HLA* human leukocyte antigen, *MN* mismatch number, *ATG* anti-thymocyte globulin

### Comparison of clinical outcomes of kidney transplant recipients according to the stage of AKI in corresponding DDs

**Fig. 6:**
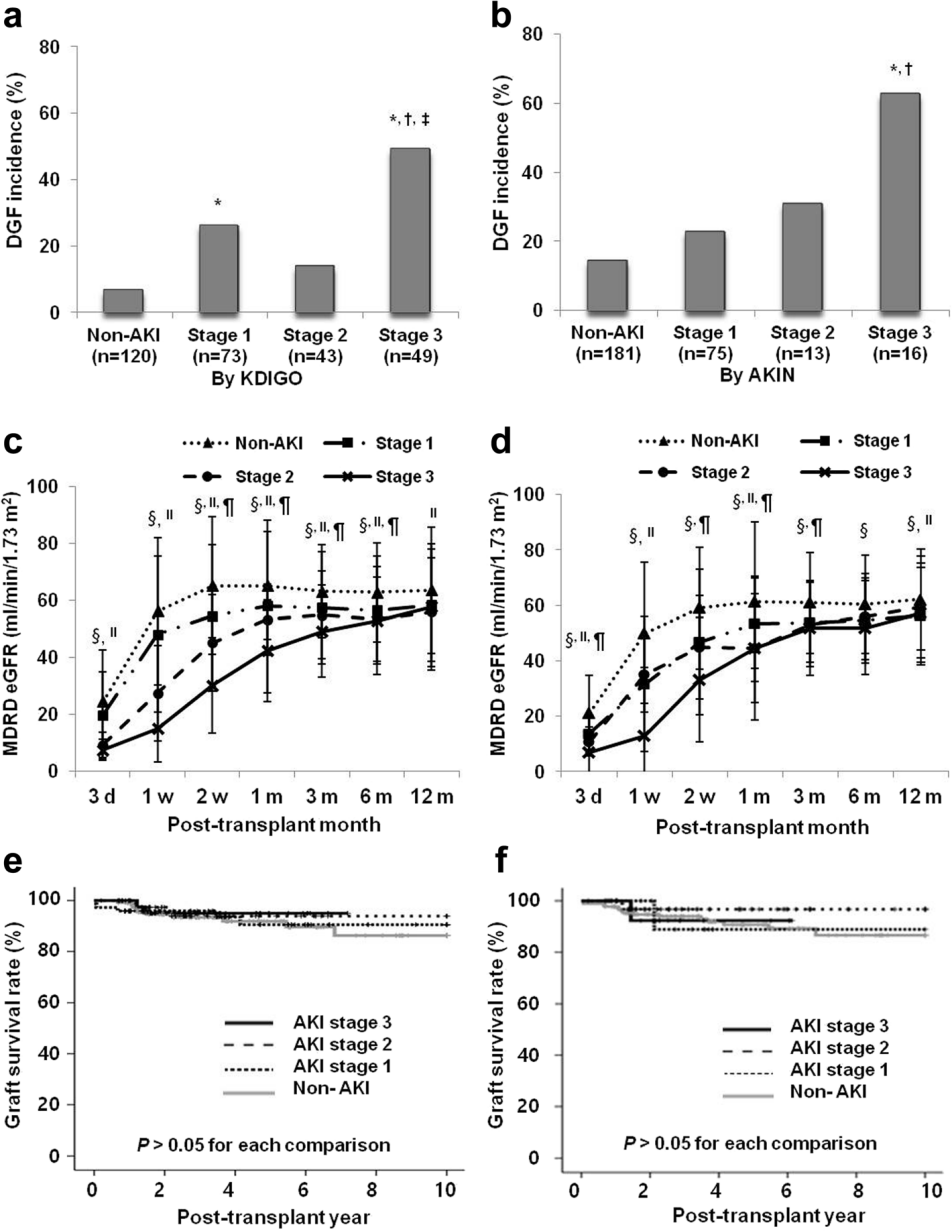
Clinical outcomes of kidney transplant recipients according to the AKI stage by either the KDIGO or AKIN criteria in corresponding donors. The incidence of DGF was highest in stage 3 AKI by either the (**a**) KDIGO or (**b**) AKIN criteria. The changing patterns of allograft function in each AKI stage group according to the AKI stage in corresponding donors by the (**c**) KDIGO or (**d**) AKIN criteria. Please note that allograft function was lowest in the stage 3 AKI group and highest in the non-AKI group in both analyses by 6 months after KT. But these differences disappeared at 1 year from KT. The allograft survival rate in each AKI stage group according to the AKI stage in corresponding donors by the (**e**) KDIGO or (**f**) AKIN criteria. Please note that there was no significant difference in long term allograft survival rate among each AKI stage group in both analyses. MDRD, the modification of diet in renal disease; eGFR, estimated glomerular filtration rate; DGF, delayed graft function; AKI, acute kidney injury; KDIGO, kidney disease: improving global outcome; AKIN, acute kidney injury network; d, day; m, month. ^*^ P <0.05 compared with non-AKI, ^†^ P < 0.05 compared with AKI stage 1, ^‡^ P <0.05 compared with non-AKI, ^§^ P <0.05 AKI stage 1 compared with non-AKI, ^II^ P <0.05 AKI stage 3 compared with non-AKI, ^¶^ P <0.05 AKI stage 3 compared with non-AKI

Based on the KDIGO criteria, DGF developed the most frequently in the stage 3 AKI group (49% (24/49), *P* < 0.05 vs. stage 1, *P* < 0.05 vs. stage 2) followed by the stage 1 group (26% (19/73)) (Fig. 6a). Based on the AKIN criteria, the rate of DGF development was also the highest in the stage 3 AKI group (62.5% (10/16), *P* < 0.05 vs. stage 1 (22.7% (17/75), *P* < 0.05 vs. stage 2 (30.8% (4/13)) (Fig. 6b). On comparison of allograft function, it showed a stage dependently deteriorating pattern upto 6 months from KT in both analyses using the KDIGO or AKIN criteria. However, these differences in allograft function according to the AKI stage nearly disappeared at 1 year from KT in both analyses (Fig. 6c, d). Also, the allograft survival rate did not show a significant difference among each AKI stage group and the non-AKI group according to either the KDIGO or AKIN criteria (*P* > 0.05 for each comparison, Fig. 6e, f).

## Discussion

In this study, we compared two standardized criteria for the diagnosis of AKI; the KDIGO and AKIN criteria in the prediction of DGF development and also other clinical outcomes in KTRs. When they were applied for the diagnosis of AKI in DDs, the KDIGO criteria had a superior predictive value for the development of DGF in corresponding KTRs in comparison with the AKIN criteria. However, this higher accuracy for DGF did not result in a better prediction of long-term allograft outcomes.

First, we compared the KDIGO criteria with the AKIN criteria in terms of the detection rate of AKI in DDs, and a significant discrepancy was found between the two criteria. According to the KDIGO criteria, additional 39 DDs were diagnosed as having AKI, which resulted in a higher incidence of AKI (54.6% (106/205)) in comparison with that using the AKIN criteria (35.6% (73/205)). In addition, a considerable discrepancy was detected in the distribution of AKI stage between the two criteria. AKI diagnosis by KDIGO showed a higher stage distribution compared to that of the AKI stage according to the AKIN criteria (*P* = 0.001). Indeed, in non-AKI patients according to the AKIN criteria, nearly 30% was defined as AKI by the KDIGO criteria, and even more, 5% was defined as stage 3 AKI (Fig. 2).

These discrepancies between the KDIGO and AKIN criteria in the diagnosis of AKI in DDs may have resulted from the differences in the details between two criteria. Although both criteria are commonly based on the change in serum creatinine and urine output, some differences exist in detailed components between the two criteria. For example, in the KDIGO criteria, AKI was defined as an increase in SCR to ≥1.5 times baseline, which is known or presumed to have occurred within the prior 7 days. In contrast, increase in SCR by ≥0.3 mg/dl within the 48 h time constraint was necessary for the diagnosis of AKI by the AKIN criteria [16, 22]. Therefore, use of the KDIGO criteria can detect AKI in more patients in comparison with the AKIN criteria as shown in a previous study performed in AKI populations [17].

Our next aim was to investigate whether a different detection rate of AKI by the KDIGO criteria can result in a difference in the prediction of DGF in corresponding KTRs. The incidence of DGF showed a significant increase in the AKI group compared to the non-AKI group, irrespective of the criteria applied in DDs (Fig. 3a, b), which is fully consistent with many previous studies [3, 5–7, 23, 24]. However, we mainly were interested in the 61 KTRs who were diagnosed as having AKI by the KDIGO criteria but not by the AKIN criteria (the AKI by KDIGO only group), because the result in this group may differentiate the predictive value for DGF between the KDIGO and AKIN criteria. Interestingly, the incidence of DGF in the AKI by KDIGO only group was very similar to that in the AKI by both criteria group and it was significantly higher than that in the non-AKI by both criteria group. This suggests that the kidney functions of patients in the AKI by KDIGO only group were more alike to those of the AKI by both criteria group rather than the non-AKI by both criteria group.

In addition, in the multivariate analysis using logistic regression analysis, detection of AKI either by KDIGO or AKIN in DDs was independently associated with DGF in corresponding KTRs. In further analysis using ROC analysis, both criteria significantly predicted DGF in corresponding KTRs. However, area under the curve in ROC analysis was significantly larger in the KDIGO criteria than in the AKIN criteria. This is consistent with the above findings, which showed that the incidence of DGF in the AKI by KDIGO only group was more alike to that of the AKI by both criteria group rather than that of the non-AKI by both criteria group. All our results suggest that the KDIGO criteria may be better in discriminating kidneys which will result in DGF in comparison with the AKIN criteria in DDs.

Third, allograft function showed a worse value during post-transplant 6 months in the AKI group in comparison with the non-AKI group in both analyses using either the KDIGO or AKIN criteria. However, this difference disappeared at one year from KT, similar to that in our previous report [5]. In three group analysis, allograft function was inferior in the AKI by both criteria group in comparison with the non-AKI by both criteria group, but this difference also disappeared at 1 year from KT (Fig. 4c). Interestingly, the AKI by KDIGO only group showed a very similar pattern of allograft function to that in the AKI by both criteria group, which is consistent with the result about DGF development. On comparison of the long-term allograft survival rate, no significant difference was detected between the two groups or among the three groups, irrespective of the applied AKI criteria as for the comparison of the 1 year allograft function in each group. Finally, diagnosis of AKI either by AKIN or KDIGO in DDs was not a significant risk factor for allograft failure in a Cox regression hazard model.

Meanwhile, the development of DGF was independently associated with allograft failure along with acute rejection and recipient BMI as in many previous studies [25–27]. This suggests that even though a high incidence of DGF in KT from an AKI donor did not result in an adverse allograft outcome, DGF itself is still an important risk factor. The exact reason for this is unclear, but it may be because the development of DGF in the non-AKI group is associated with worse allograft outcomes. The development of DGF in non-AKI kidneys from DDs is frequently due to acute rejection not due to acute tubular necrosis, which may explain the poor allograft outcome in those cases [6, 28]. However, further investigation is required to clarify this issue.

Lastly, we investigated whether the severity of AKI in DDs has a significant impact on the clinical outcomes of corresponding KTRs. As expected, stage 3 AKI in DDs resulted in the highest incidence of DGF and a lower allograft function during post-transplant 6 months in corresponding KTRs compared to that in stage 1 or 2 AKI in DDs in both analyses using the KDIGO or AKIN criteria. However, these differences showed a decreasing pattern in allograft function at one year from KT, and long term allograft survival rate did not show significant differences in comparison across the AKI stage both by the KDIGO or AKIN criteria. This suggests that not only AKI diagnosis but also the severity of AKI in DDs may not have a significant impact on long-term allograft outcomes in corresponding KTRs.

Our study has some limitations. Firstly, not all recipients corresponding to the donors included in this study were included in this analysis because some organs were transferred to another institution according to the organ distribution rule in Korea, which could have induced a bias during analysis. Secondly, this study was performed as a retrospective chart review. A prospective multicenter study conducted in a larger patient group is required to overcome the above issues.

## Conclusions

In conclusion, the KDIGO criteria may be more appropriate than the AKIN criteria in terms of the prediction of DGF in corresponding KTRs. However, AKI or AKI stage by any criteria failed to affect long-term allograft outcomes, which suggests that AKI itself may not be a reason for exclusion from kidney donation in DDKT.

## Abbreviations

AKI: Acute kidney injury
AKIN: Acute kidney injury network
DDKT: Deceased donor kidney transplantation
DDs: Deceased donors
DGF: Delayed graft function
HLA: Human leukocyte antigen
KDIGO: Kidney Disease: Improving Global Outcomes
KT: Kidney transplantation
MDRD: Modification of diet in renal disease
PRAs: Panel-reactive antibodies
RIFLE: Risk, Injury, Failure, Loss, End-Stage Kidney Disease
ROC: Receiver Operating Characteristics
RRT: Renal replacement therapy
SCR: Serum creatinine
